# Supplementary material for: Add and Go: FRET Acceptor for Live-Cell Measurements Modulated by Externally Provided Ligand
Source: Int J Mol Sci. 2022 Apr 15;23(8):4396. doi: 10.3390/ijms23084396 (PMC9026985; doi:10.3390/ijms23084396)
Supplement: Supplementary file 1 [file ijms-23-04396-s001.zip › Supplementary materials.pdf]

## Supplementary materials

Add and Go: FRET Acceptor for Live-Cell Measurements Modulated by Externally Provided Ligand

Alexey S. Gavrikov[1], Nina G. Bozhanova[1], Mikhail S. Baranov[1, 2], Alexander S. Mishin[1*]
[1] Shemyakin-Ovchinnikov Institute of Bioorganic Chemistry, Russian Academy of Sciences, Miklukho-Maklay st. 16/10, Moscow, 117997, Russia
[2] Pirogov Russian National Research Medical University, Ostrovitianov 1, Moscow, 117997, Russia
[*]correspondence: mishin@ibch.ru

## 1. Supplementary materials and methods

*1.1 Constructs sequences*

>H2B-mNeonGreen-DiB1
MPEPAKSAPAPKKGSKKAVTKAQKKGGKKRKRSRKESYSIYVYKVLKQVHPDTGISS
KAMGIMNSFVNDIFERIAGEASRLAHYNKRSTITSREIQTAVRLLLPGELAKHAVSEGT
KAITKYTSAKDPPVATMVSKGEEDNMASLPATHELHIFGSINGVDFDMVGQGTGNPN
DGYEELNLKSTKGDLQFSPWILVPHIGYGFHQYLPYPDGMSPFQAAMVDGSGYQVH
RTMQFEDGASLTVNYRYTYEGSHIKGEAQVKGTGFPADGPVMTNSLTAADWCRSKK
TYPNDKTIISTFKWSYTTGNGKRYRSTARTTYTFAKPMAANYLKNQPMYVFRKTELK
HSKTELNFKEWQKAFTDVMGMDELYKGDPPVATMASSPTPPRGVTVVNNFDCKRYL
GTWYEIARFDHRFERGLEKVTATYSLRDDGGLNVINKGYNPDRGMWQQSEGKAYFT
GAPTRAALKVSFFGPFYGGYNVIALDREYRHALVCGPDRDYLWINSRTPTISDEVKE
MLAVATREGFDVSKFIWVQQPGSGS

>Vimentin-DiB1
MSTRSVSSSSYRRMFGGPGTASRPSSSRSYVTTSTRTYSLGSALRPSTSRSLYASSPGG
VYATRSSAVRLRSSVPGVRLLQDSVDFSLADAINTEFKNTRTNEKVELQELNDRFANY
IDKVRFLEQQNKILLAELEQLKGQGKSRLGDLYEEEMRELRRQVDQLTNDKARVEVE
RDNLAEDIMRLREKLQEEMLQREEAENTLQSFRQDVDNASLARLDLERKVESLQEEI
AFLKKLHEEEIQELQAQIQEQHVQIDVDVSKPDLTAALRDVRQQYESVAAKNLQEAE
EWYKSKFADLSEAANRNNDALRQAKQESTEYRRQVQSLTCEVDALKGTNESLERQ
MREMEENFAVEAANYQDTIGRLQDEIQNMKEEMARHLREYQDLLNVKMALDIEIAT
YRKLLEGEESRISLPLPNFSSLNLRETNLDSLPLVDTHSKRTLLIKTVETRDGQVINETS
QHHDDLEGDPPVATGMASSPTPPRGVTVVNNFDCKRYLGTWYEIARFDHRFERGLE
KVTATYSLRDDGGLNVINKGYNPDRGMWQQSEGKAYFTGAPTRAALKVSFFGPFYG
GYNVIALDREYRHALVCGPDRDYLWINSRTPTISDEVKEMLAVATREGFDVSKFIWV
QQPGSGS

>Tension sensor
MPVFHTRTIESILEPVAQQISHLVIMHEEGEVDGKAIPDLTAPVSAVQAAVSNLVRVGK
ETVQTTEDQILKRDMPPAFIKVENACTKLVRAAQMLQADPYSVPARDYLIDGSRGILS
GTSDLLLTFDEAEVRKIIRVCKGILEYLTVAEVVETMEDLVTYTKNLGPGMTKMAKM
IDERQQELTHQEHRVMLVNSMNTVKELLPVLISAMKIFVTTKNTKSQGIEEALKNRNF
TVEKMSAEINEIIRVLQLTSWDEDAWASKDTEAMKRALALIDSKMNQAKGWLRDPN
APPGDAGEQAIRQILDEAGKAGELCAGKERREILGTCKTLGQMTDQLADLRARGQG
ATPMAMQKAQQVSQGLDLLTAKVENAARKLEAMTNSKQAIAKKIDAAQNWLADPN
GGSEGEEHIRGIMSEARKVAELCEEPKERDDILRSLGEISALTAKLSDLRRHGKGDSPE
ARALAKQIATSLQNLQSKTNRAVANTRPVKAAVHLEGKIEQAQRWIDNPTVDDRGV
GQAAIRGLVAEGRRLANVMMGPYRQDLLAKCDRVDQLAAQLADLAARGEGESPQA
RAIAAQLQDSLKDLKARMQEAMTQEVSDVFSDTTTPIKLLAVAATAPSDTPNREEVFE
ERAANFENHAARLGATAEKAAAVGTANKTTVEGIQATVKSARELTPQVVSAARILLR
NPGNQAAYEHFETMKNQWIDNVEKMTGLVDEAIDTKSLLDASEEAIKKDLDKCKVA
MANMQPQMLVAGATSIARRANRILLVAKREVENSEDPKFREAVKAASDELSKTISPM
VMDAKAVAGNISDPGLQKSFLDSGYRILGAVAKVREAFQPQEPDFPPPPPDLEHLHLT
DELAPPKPPLPEGEVPPPRPPPPEEKDEVEMVSKGEEDNMASLPATHELHIFGSINGVD
FDMVGQGTGNPNDGYEELNLKSTKGDLQFSPWILVPHIGYGFHQYLPYPDGMSPFQ
AAMVDGSGYQVHRTMQFEDGASLTVNYRYTYEGSHIKGEAQVKGTGFPADGPVMT
NSLTAADWCRSKKTYPNDKTIISTFKWSYTTGNGKRYRSTARTTYTFAKPMAANYLK
NQPMYVFRKTELKHSKTELNFKEWQKAFTDVMGMDELYKGPGGAGPGGAGPGGA
GPGGAGPGGAGPGGAGPGGAGPGGAMASSPTPPRGVTVVNNFDCKRYLGTWYEIA
RFDHRFERGLEKVTATYSLRDDGGLNVINKGYNPDRGMWQQSEGKAYFTGAPTRAA
LKVSFFGPFYGGYNVIALDREYRHALVCGPDRDYLWINSRTPTISDEVKQEMLAVATR
EGFDVSKFIWVQQPGSAAAGEFPEQKAGEAINQPMMMAARQLHDEARKWSSKGND
IIAAAKRMALLMAEMSRLVRGGSGNKRALIQCAKDIAKASDEVTRLAKEVAKQCTD
KRIRTNLLQVCERIPTISTQLKILSTVKATMLGRTNISDEESEQATEMLVHNAQNLMQS
VKETVREAEAASIKIRTDAGFTLRWVRKTPWYQGS

>YAP-mNeonGreen
MDPGQQPPPQPAPQGQGQPPSQPPQGQGPPSGPGQPAPAATQAAPQAPPAGHQIVHV
RGDSETDLEALFNAVMNPKTANVPQTVPMRLRKLPDSFFKPPEPKSHSRQASTDAGT
AGALTPQHVRAHSSPASLQLGAVSPGTLTPTGVVSGPAATPTAQHLRQSSFEIPDDVPL
PAGWEMAKTSSGQRYFLNHIDQTTTWQDPRKAMLSQMNVTAPTSPPVQQNMMNSA
SGPLPDGWEQAMTQDGEIYYINHKNKTTSWLDPRLDPRFAMNQRISQSAPVKQPPPL
APQSPQGGVMGGSNSNQQQQMRLQQLQMEKERLRLKQQELLRQAMRNINPSTANS
PKCQELALRSQLPTLEQDGGTQNPVSSPGMSQELRTMTTNSSDPFLNSGTYHSRDEST
DSGLSMSSYSVPRTPDDFLNSVDEMDTGDTINQSTLPSQQNRFPDYLEAIPGTNVDLG
TLEGDGMNIEGEELMPSLQEALSSDILNDMESVLAATKLDKESFLTWLDPPVATMVS
KGEEDNMASLPATHELHIFGSINGVDFDMVGQGTGNPNDGYEELNLKSTKGDLQFSP
WILVPHIGYGFHQYLPYPDGMSPFQAAMVDGSGYQVHRTMQFEDGASLTVNYRYTY
EGSHIKGEAQVKGTGFPADGPVMTNSLTAADWCRSKKTYPNDKTIISTFKWSYTTGN
GKRYRSTARTTYTFAKPMAANYLKNQPMYVFRKTELKHSKTELNFKEWQKAFTDV
MGMDELYKG

>14-3-3-DiB1
MERASLIQKAKLAEQAERYEDMAAFMKGAVEKGEELSCEERNLLSVAYKNVVGGQ
RAAWRVLSSIEQKSNEEGSEEKGPEVREYREKVETELQGVCDTVLGLLDSHLIKEAG
DAESRVFYLKMKGDYYRYLAEVATGDDKKRIIDSARSAYQEAMDISKKEMPPTNPIR
LGLALNFSVFHYEIANSPEEAISLAKTTFDEAMADLHTLSEDSYKDSTLIMQLLRDNL
TLWTADNAGEEGGEAPQEPQSDPPVATMASSPTPPRGVTVVNNFDCKRYLGTWYEIA
RFDHRFERGLEKVTATYSLRDDGGLNVINKGYNPDRGMWQQSEGKAYFTGAPTRAA
LKVSFFGPFYGGYNVIALDREYRHALVCGPDRDYLWINSRTPTISDEVKQEMLAVATR
EGFDVSKFIWVQQPGSGSG

**2. Supplementary figures and movies**

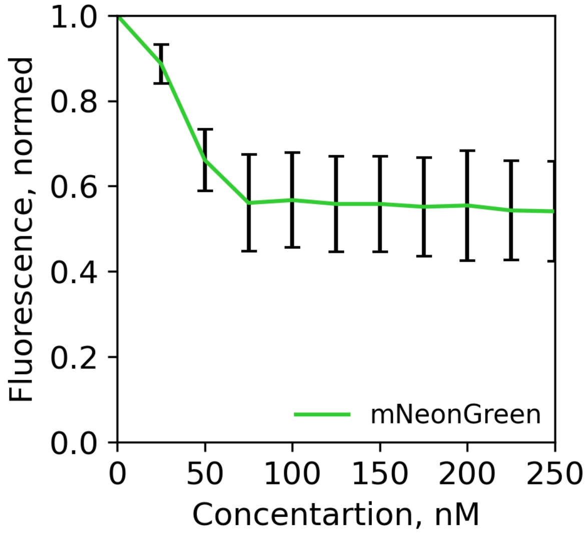


**Figure S1**. **Live-cell titration of modulated FRET acceptor.** Fluorescence intensity of mNeonGreen in live HeLa cells transiently transfected with H2B-mNeonGreen-DiB1 in the presence of different concentrations of compound **3**; error bars represent standard deviation (technical replicates = 17).

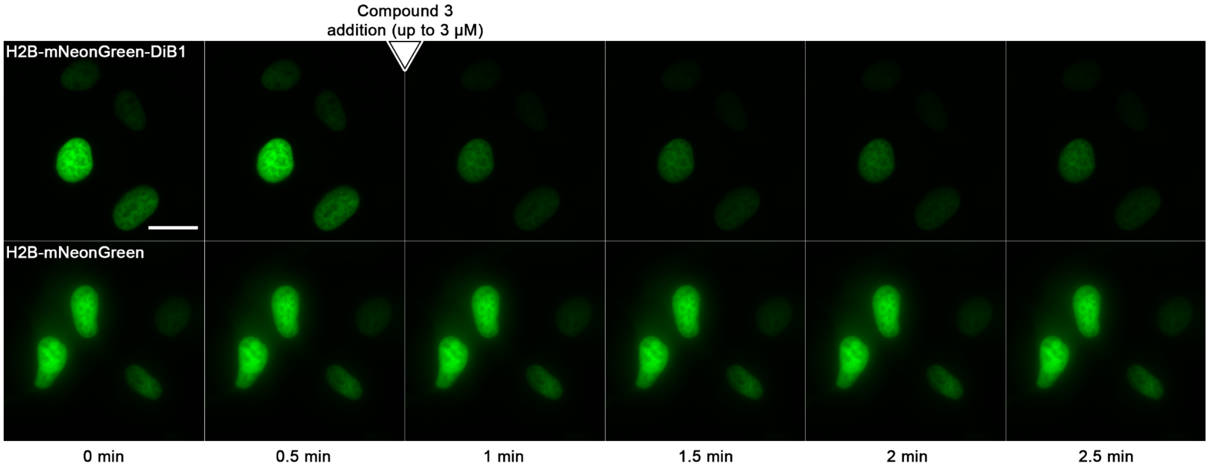


**Figure S2**. **Live-cell image sequence for FRET acceptor activation.** Widefield fluorescence imaging of live HeLa Kyoto cells transiently transfected with H2B-mNeonGreen (lower row) and H2B-mNeonGreen-DiB1 (upper row) constructs before and after compound **3** addition up to 3 μM concentration; Scale bar is 20 μm.

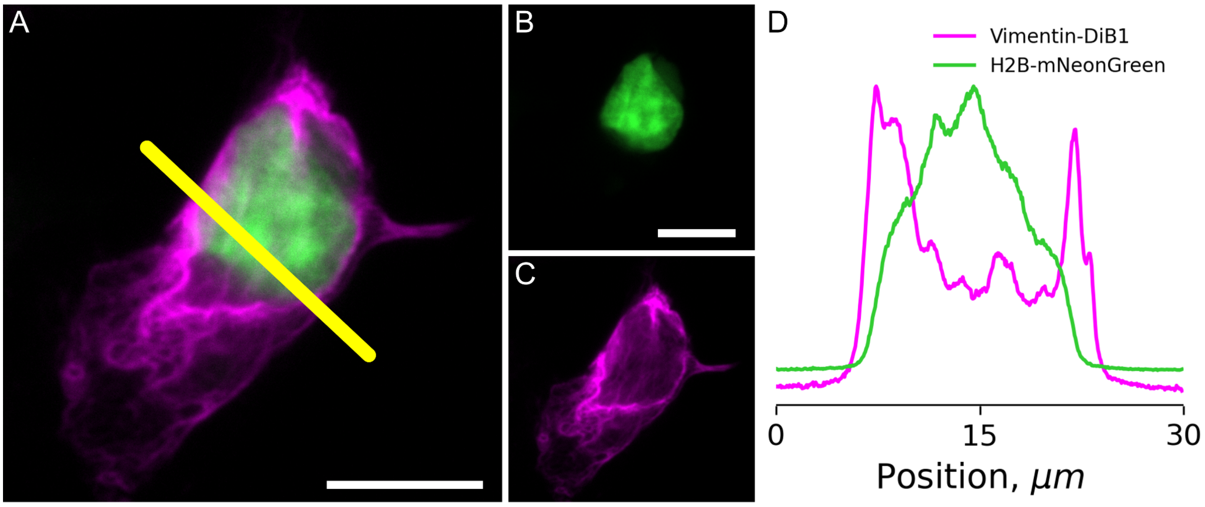


**Figure S3**. **Imaging of non-interacting proteins labeled with modulated FRET pair.** Live-cell imaging of DiB1 and mNeonGreen on non-interacting proteins. Widefield fluorescence imaging of live HeLa Kyoto cells transiently cotransfected with H2B-mNeonGreen and vimentin-DiB1 in the presence of 100 nM of compound **3**. (**A**) Composed image of labeled nucleus and vimentin filaments. (**B**, **C**) Separate channels of panel (**A**). (**D**) Fluorescence intensity profile (indicated by yellow line on panel (**A**)); Scale bars are 20 µm.

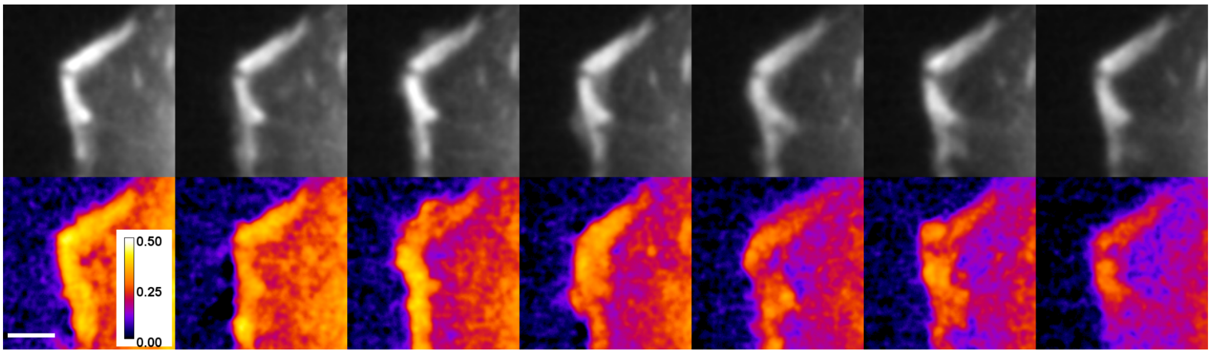


**Figure S4**. **Tension sensor with single-channel FRET detection.** Widefield fluorescence imaging of dynamic tension changes in focal contacts of live HeLa Kyoto cells transiently transfected with tension sensor construct. Frame interval – 6 minute. A cyclic FRET acceptor activation (staining with compound **3**, followed by washing with HHBS buffer) was performed on every time point. Upper row – widefield images before staining, lower row – FRET efficiency images; pseudocolor indicates FRET efficiency after the addition of 3 µM compound **3**; Scale bar is 3 µm.

**Supplementary Movie S1. Cyclic FRET activation.** Repeated cycles of FRET activation in live HeLa cells transiently transfected with H2B-mNeonGreen-DiB1 construct. Green lines represent average intensities of mNeonGreen (donor) fluorescence in labeled nuclei. Acceptor activation was achieved by the addition of HHBS buffer supplied with 3 µM of compound **3**. Washing was performed with HHBS buffer; Scale bar is 10 µm. Video plays at 30 fps.

**Supplementary Movie S2**. **Tension sensor with single-channel FRET detection.** Widefield fluorescence imaging of dynamic tension changes in focal contacts of live HeLa Kyoto cells transiently transfected with tension sensor construct. Frame interval - 6 minutes. A cyclic FRET acceptor activation (staining with compound **3**, followed by washing with HHBS buffer) was performed on every time point. Upper image - widefield microscopy, lower image - FRET efficiency image; pseudocolor indicates FRET efficiency after the addition of 3 µM compound 3; Scale bar is 3 µm. Video plays at 2 fps.
